# The *Salmonella* pathogenicity island 13 contributes to pathogenesis in streptomycin pre-treated mice but not in day-old chickens

**DOI:** 10.1186/s13099-016-0098-0

**Published:** 2016-05-02

**Authors:** Jacob R. Elder, Kim Lam Chiok, Narayan C. Paul, Gary Haldorson, Jean Guard, Devendra H. Shah

**Affiliations:** Department of Veterinary Microbiology and Pathology, Washington State University, Pullman, WA 99164-7040 USA; Paul Allen School for Global Animal Health, College of Veterinary Medicine, Washington State University, Pullman, WA 99164-7040 USA; Egg Quality and Safety Research Unit, Agriculture Research Service, United States Department of Agriculture, Athens, GA 30605 USA

**Keywords:** *Salmonella*, *Salmonella* pathogenicity island, *Salmonella* Enteritidis, Pathogenesis, SPI-13, Host-specificity, Chicken, Streptomycin pre-treated mice

## Abstract

**Background:**

*Salmonella enterica* serovar Enteritidis (*S.* Enteritidis) is a human and animal pathogen that causes gastroenteritis characterized by inflammatory diarrhea and occasionally an invasive systemic infection. *Salmonella* pathogenicity islands (SPIs) are horizontally acquired genomic segments known to contribute to *Salmonella* pathogenesis. The objective of the current study was to determine the contribution of SPI-13 to *S.* Enteritidis pathogenesis.

**Methods:**

We deleted the entire SPI-13 (∆SPI-13) from the genome of *S*. Enteritidis CDC_2010K_0968 strain isolated from a human patient during the 2010 egg-associated outbreak in the US. The kinetics of infection of the wild-type parent and the ∆SPI-13 were compared in orally challenged day-old chickens and streptomycin pre-treated mice. The degree of intestinal inflammation and the survival of mutant strain within the avian (HD11) and murine (RAW264.7) macrophages were also determined.

**Results:**

The deletion of the SPI-13 resulted in impaired infection kinetics of *S.* Enteritidis in streptomycin pre-treated mice which was characterized by significantly lower (*P* < 0.05) viable counts in the ceca, liver and spleen, impaired ability to induce intestinal inflammation and reduced survival within murine macrophages. Conversely, there were no significant differences in the infection kinetics of ∆SPI-13 in day-old chickens in any of the organs tested and the survival of ∆SPI-13 within chicken macrophages remained unaltered.

**Conclusions:**

The results of this study show that SPI-13 contributes to the pathogenesis of *S.* Enteritidis in streptomycin pre-treated mice but not in day-old chickens and raises the possibility that SPI-13 may play a role in pathogenesis and the host adaptation/restriction of *Salmonella* serovars.

## Background

*Salmonella enterica* subspecies *enterica* serovar Enteritidis (*S.* Enteritidis) is a major food-borne pathogen that causes inflammatory diarrhea in immunocompetent patients, however poor immune response or co-infection with malaria or HIV may result in invasive infections with severe systemic illness [1, 2]. The *Salmonella* pan-genome has 23 annotated genomic islands [3, 4] that are referred to as *Salmonella* pathogenicity islands (SPIs). Of these, SPI-1 and SPI-2 have been extensively characterized. The current paradigm is that SPI-1 is required for invasion of the epithelial cells in the intestinal tract whereas SPI-2 is required for survival in macrophages and systemic spread (Reviewed in [5]). However, the majority of the other SPIs are poorly characterized and their contribution to the biology of *Salmonella* in general and pathogenesis in particular remains unclear.

**Fig. 1:**
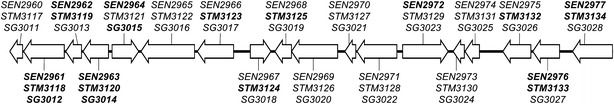
Genetic organization of SPI-13 in *S.* Enteritidis P125109 [13], *S.* Typhimurium LT2 [34] and *S.* Gallinarum 287/91 [13]. Gene names are listed for P125109 (*top*, *SEN*—), LT2 (*middle*, *STM*—) and 287/91 (*lower*, *SG*—). *Bold* gene names indicate where transposon mutagenesis has resulted in in vivo fitness defects in chickens or mice [6, 8, 11]

SPI-13 was originally identified in *S.* Gallinarum (an avian host-adapted serotype) by employing a negative selection screening approach which has been widely used to identify genes that may contribute to in vivo fitness of *Salmonella* [6]. In this approach, large numbers of mutants are pooled and screened for in vivo fitness defects (indicated by loss or lower recovery of a mutant) using animal or cell culture models. In the previous study, we screened a library of >4000 *S.* Gallinarum (an avian host-adapted serotype) transposon insertion mutants in orally challenged day-old chickens and demonstrated that mutations within three genes (*SG3012, SG3014*–*SG3015*) resulted in reduced organ colonization and competitive defects in vivo [6]. Subsequent mapping and bioinformatics analysis of these genes resulted in identification of SPI-13 (Fig. 1) which includes eighteen open reading frames (ORFs) representing a region adjacent to tRNA *pheV* with a G + C content (48.1 mol%) significantly lower than the mean G + C content (52 mol%) of the *Salmonella* genome.

The sequences downstream of tRNA genes are hotspots for recombination in bacteria, it is therefore not surprising that SPI-13 appears to have undergone multiple recombination events in the evolution of the genus *Salmonella*. Evidence of recombination events at this locus include insertion/deletion of segments of SPI-13 resulting in distinct SPI-13 sequences observed between *S. enterica* and *S. bongori*, as well as differences between subspecies and serovars within *S. enterica. Salmonella bongori* shares very few SPI-13 genes with *S.* Enteritidis and strains representing the subspecies *indica*, *salamae*, *diarizonae, arizonae* and *houtenae* also lack many SPI-13 genes [7]. The differences in gene content between *S. enterica* subspecies could be related to host adaptation as *S. enterica* subspecies *enterica* is associated with warm-blooded host while *S. bongori* and the other *Salmonella* subspecies are associated with cold-blooded hosts. Interestingly, there are also SPI-13 gene content differences within subspecies *enterica* serovars that seem to be related to host adaptation. Serovars Typhi, Paratyphi A and Sendai, that cause typhoid-like disease and are human-adapted, have a different SPI-13 gene composition compared to the majority of the non-typhoidal *Salmonella* (NTS) serovars with broad host range [6, 7]. *Klebseilla pneumoniae* and *Yersinia pestis* have homologs to some of the genes in SPI-13 which also suggests that SPI-13 is not monophyletic.

Since the original identification of SPI-13 in an avian host-adapted *S.* Gallinarum, few negative selection screens have been conducted using non-host restricted NTS serovars in which several SPI-13 genes were identified. Chaudhuri et al. [8] screened a library containing pools of >7700 mutants of *S.* Typhimurium in orally infected chickens, pigs, and calves and reported that insertion mutations in up to fifteen genes of SPI-13 resulted in negative selection of these mutants in the intestines of these hosts. Moreover, few SPI-13 mutants of *S.* Typhimurium were negatively selected in internal organs in intra-peritoneally [8, 9] and orally inoculated mice [10]. To date, only one study reported use of *S.* Enteritidis as a model organism in which insertion mutations in seven SPI-13 genes (*SEN2961*–*SEN2964*, *SEN2972*, *SEN2976*–*SEN2977*) resulted in negative selection in intra-peritoneally inoculated mice [11]. It is important to note that the negative selection of a mutant during such large-scale in vivo screening assays could result from number of underlying factors. These include direct impact of the mutation on the pathogen-host cell interaction or indirectly due to in vivo competitive growth defects or because of impaired metabolic fitness or merely due stochastic loss of a mutant from a population. Moreover, the negative selection-screening assays employed in different studies are often limited to a single organ and a single time point and only few have used natural route of infection. Thus, although negative selection of SPI-13 mutants reported in the published studies raises a possibility that SPI-13 may have a role in pathogenesis of *Salmonella,* this has not been conclusively demonstrated.

The specific objective of this study was to directly demonstrate the role of SPI-13 in *S.* Enteritidis pathogenesis by constructing *S.* Enteritidis mutant lacking the entire SPI-13 and determining the effects of absence of SPI-13 on kinetics of infection (i.e. intestinal colonization and invasion, modulation of gut inflammation and internal organ colonization). To dissect the role of SPI-13 in pathogenesis, we used two biologically relevant animal models, which included: (1) streptomycin pre-treated mouse (an established model for human intestinal disease) and (2) day-old chickens (the reservoir host and a major source of human infection). To the best of our knowledge, this is the first study showing the direct evidence that SPI-13 contributes to intestinal pathogenesis of *S.* Enteritidis in streptomycin pre-treated mice. The results of this study also point towards the possibility that SPI-13 is likely involved in host- adaptation and propagation of *S.* Enteritidis in gastrointestinal environment of mice, but not in chickens.

## Methods

### Bacterial strains and growth media

The *S.* Enteritidis CDC_2010K_0968 strain, isolated from a human patient in Ohio during the 2010 egg-associated outbreak [12], was used as the wild-type (WT) parent strain for constructing following mutants: (1) a k/o mutant of SPI-13 (ΔSPI-13), (2) a k/o mutant of SPI-14 (ΔSPI-14), (3) a k/o mutant of both SPI-13 and SPI-14 (ΔSPI-13/14) and, (4) a kanamycin resistant derivative of the WT strain (WT Kan^R^). The ΔSPI-14 and ΔSPI-13/14 mutants were included as control strains to compare and confirm the role of SPI-13 in pathogenesis of *S.* Enteritidis in streptomycin pre-treated mouse model (see below). The kanamycin resistant derivative of the reference strain P125109 (UK Kan^R^) was also included for comparison of infection kinetics in streptomycin pre-treated mice [13]. The λ-Red recombinase system was used to construct ΔSPI-13, ΔSPI-14, ΔSPI-13/14 and Kan^R^ WT strains following procedures described previously [14]. For the ΔSPI-13 strain the entire SPI-13 was replaced with the kanamycin resistance cassette encoded on the pKD4 plasmid amplified with the forward primer, SPI13KOFw (5′-TATAAACGGATGCGTGATCATAATAAAGGCAGTAATAGTAAGTTTTAACAGTGTAGGCTGGAGCTGCTTC-3′), and reverse primer, SPI13KORv (5′-CGCTACAGGTCAGACGGCGCGGAGCTAATGTTTTTTAACGAGGCTTTATCATATGAATATCCTCCTTAG-3′). The ΔSPI-14 strain was generated by replacement of SPI-14 with the kanamycin resistant cassette using the forward primer, SPI14KOFw (5′-TTTTAAGATATATTGAATTATCAGATGCTCCATTCAAATGAGAGACGAGAGTGTAGGCTGGAGCTGCTTC-3′), and the reverse primer SPI14KORv (5′-TGCATAACATGGATAAAATGGGTAGTCATGCTAGCGAGATAAGACAATGACATATGAATATCCTCCTTAG-3′). The ΔSPI-13/14 mutant was constructed by replacing SPI-13 in the previously constructed ΔSPI-14 mutant with the chloramphenicol resistance cassette using the primers listed above for SPI-13 k/o. For the construction of WT Kan^R^ and UK Kan^R^ strains, the kanamycin resistance cassette was amplified using the forward primer, att_tn7KOFw (5′-AGCGCAGGTAGGCGTAGCACCTCTTAGTCGCTCTTCAGCCACCATAGAGAGTGTAGGCTGGAGCTGCTTC-3′), and reverse primer, att_tn7KORv (5′-GGCCGTCGATAGACGGCCTTTTTTTGTGCGCCGTGACAGGCGCTGTTCTTATATGAATATCCTCCTTA-3′). This cassette was inserted at the *tn7* attachment site (*att_tn7,* found between nucleotides 3,939,373 and 3,939,408) which lies in the intergenic region between *SEN3674* and *glmS* in the *S*. Enteritidis strain P125109 genome (NCBI GenBank accession: NC_011294). Unless otherwise noted, *S.* Enteritidis and *E. coli* strains were grown in Luria–Bertani (LB) broth (Difco, USA) overnight (16 h) at 37 °C with shaking at 200 rpm. When appropriate, the medium was supplemented with 1.6 % (w/v) Bacto Agar (Difco), carbenicillin (Cb, 100 μg/ml), chloramphenicol (Cm, 20 μg/ml) and kanamycin (Km, 50 μg/ml).

### Mouse infection experiments

All animal experiments were performed following the protocols approved by Institutional Animal Care and Usage Committee (IACUC). The streptomycin pre-treated mouse model was used as previously described [15]. For each experiment, 7–8 week-old, female C57BL/6 mice were acquired from Harlan laboratories, USA. Food was withheld for 4 h prior to orogastric administration of 20 mg of streptomycin (Sigma Aldrich, USA) in 100 µl of sterile H_2_O. At 24 h post streptomycin treatment, food was withheld for 4 h. Subsequently, mice were orogastrically challenged with ~7 log_10_ CFU WT parent (WT Kan^R^) strain or one of the ΔSPI-13, ΔSPI-14, or ΔSPI-13/14 mutant strains. At days 5 and 7 post-infection (p.i.), three mice were sacrificed. The liver, spleen, and cecum were collected for direct plate counts and section of the cecum was also collected for histopathological analysis (see below). Intracellular counts in the cecum were determined by washing the tissues 3× in sterile PBS to remove as much of the intestinal contents and extracellular bacteria as possible and then incubating for 30 min at 37 °C with gentamicin (200 µg/ml) to kill any remaining extracellular bacteria. The sections were washed with sterile PBS to remove residual gentamicin, homogenized, then treated with 0.5 % Triton-X 100 to lyse epithelial cells and release intracellular bacteria. Samples from the liver, spleen, and cecum were weighed, homogenized in sterile maximum recovery diluent (MRD, Difco), serially diluted and directly plated on XLT-4 media (Difco) with 50 μg/ml kanamycin when appropriate. Only in the cases when no colonies were recovered from samples by direct plating of organ homogenates were samples enriched; 500 µl of homogenate was used to inoculate 10 ml tetrathionate broth (TTB, Neogen) prepared according to manufacturer’s directions. Enrichment cultures were incubated at 37 °C for 24 h prior to plating on XLT-4 media followed by incubation for 24 h at 37 °C. If TTB enrichment cultures were negative after 24 h, they were plated again at 48 h. If a sample was positive after 24 or 48 h of enrichment they were assigned the limit of detection for this experiment as the lowest CFU/g observed for that specific organ across the experiment. If samples were negative at both time points 0 CFU/g was assigned for statistical analysis. Organ homogenates were directly plated on XLT-4 media (Difco) supplemented with 50 μg/ml kanamycin when appropriate. Samples found negative by direct plating were enriched as described above.

### Histological analysis

Cecal sections from mice were embedded in paraffin wax and stained with hematoxylin and eosin (H&E). The sections were analyzed independently by two individuals blinded to the sample IDs to assign total inflammation scores as described previously with minor modifications [15]. Briefly, the score for percent submucosal edema (% SE) was calculated from the proportion of the diameter of mucosa made up by the space between the tunica muscularis and the epithelial layer using following scores: 0 for no detectable edema, 1 for detectable edema (0–10 % of the diameter of the cross section of the intestinal mucosa and submucosa), 2 for moderate edema (11–40 %), and 3 for severe edema (>40 %). Infiltration of the lamina propia by polymorphonuclear (PMN) cells was scored according to number of PMNs in the lamina propia per 10 high power (400×) fields by using following scores: 0 for normal (<5), 1 for slight infiltration (6–20), 2 moderate (21–60), 3 for high infiltration (61–100), and 4 for severe (>100). Reduction in number of goblet cells were scored based on the number of goblet cells per high power field; 0 for normal (>28), 1 for slight reduction (11–28), 2 for moderate reduction (1–10), and 3 for severe reduction (<1). Integrity of the intestinal epithelium was scored as follows: 0 no detectable loss of integrity, 1 for epithelial desquamation, 2 for epithelial erosion and 3 for epithelial ulceration. Finally, a total combined score was calculated. The combined score of 0 was considered to indicate no inflammation, 1, 2 indicated minimal inflammation, 3, 4 indicated slight inflammation, 5–8 indicated significant inflammation, and 9–13 indicated severe inflammation.

### Chicken infection experiments

Specific-pathogen-free (SPF) White Leghorn eggs were acquired from Sunrise farms (Catskill, NY) and incubated for 18 days in the egg incubator (Ova-Easy 190 Advance Cabinet Incubator, Brinsea Products Inc, Titusville, FL) before transferring to a hatcher (1550 hatcher-GQF, GQF manufacturer Co, Savannah, GA) for 3 days following manufacturer’s instructions. Newly hatched SPF chicks were transferred and housed in HEPA-filtered isolator cages. Chickens were orally challenged with 8.5 log_10_ CFU of ΔSPI-13 mutant and the WT parent strain, respectively at day-1 of age [16]. Subsequently, four chickens were sacrificed at days-1, 3, 5, 7, 14 and 28 p.i. except on 5 and 28 days p.i. when three birds were sacrificed. The spleen, liver, duodenum, jejunum, ileum, and ceca were collected for direct plate counts. For the determination of viable bacterial counts in the small intestine, segments from the duodenum, jejunum and ileum were pooled for both intracellular and total counts. A portion of ceca and each segment of the small intestine were processed for determination of intracellular counts similar to as described for mouse experiments.

### Intra-macrophage survival assays

RAW264.7 mouse macrophages (ATCC) were grown in D-MEM medium (Gibco, USA) supplemented with 10 % fetal bovine serum (FBS, Sigma Aldrich, USA), 2 mM l-glutamine, and 20 μg/ml gentamicin at 37 °C with 5 % CO_2_. HD11 chicken macrophages were grown in IMDM medium (Gibco, USA) supplemented with 10 % FBS at 42 °C with 5 % CO_2_. Gentamicin-protection assays were performed as described previously [17]. Briefly, for each experiment, RAW264.7 or HD11 cells were seeded in three 48-well plates each with 1.25 × 10^5^ cells per well and grown for 16–24 h. Overnight (16 h) cultures of *S.* Enteritidis strains were sub-cultured in fresh LB medium and grown for ~3 h at 37 °C with shaking at 200 rpm until OD_600_ of 0.6 was achieved. Bacterial strains were pelleted by centrifugation at 13,000*g* for 2 min, washed three times with sterile PBS and resuspended in pre-warmed D-MEM (RAW264.7) or IMDM (HD11) to obtain 2.5 × 10^6^ CFU/100 μl. Subsequently, 100 μl of each *S.* Enteritidis strain was added to each well (multiplicity of infection of ~20), infections were synchronized by spinning the plates at 250*g* for 5 min. Cell infection was allowed to proceed for 30 min followed by three washings with PBS and gentamicin treatment (200 μg/ml) for 30 min to kill extracellular bacteria. Next, the cells from plate-1 were washed 3× with sterile PBS and lysed with 0.5 % Triton-X 100. The numbers of intracellular bacteria were enumerated via direct plate counts which served as a measure of total uptake of each bacterial strain. In plate-2 and plate-3, the media was replaced with the lower concentration of gentamicin (20 μg/ml) followed by incubation for 2 h (plate-2) and 20 h (plate-3). At 2 and 20 h post infection, cells from respective plates were processed identically as described for plate-1. Percent survival/replication of *S.* Enteritidis strains at 2 and 20 h was determined as follows: CFU at 2 or 20 h/total uptake CFU × 100. Each strain was tested in duplicates in at least three independent experiments.

### Statistical analysis

Statistically significant (*P* < 0.05) differences in mean log_10_ CFU counts in each organ were determined using Student’s *t* test for comparing the two means in the preliminary mouse experiments (for determination of dose and comparing WT strains) and the chicken experiment. A one-way ANOVA with Tukey’s post hoc was used to compare means and identify statistically significant differences between the four groups in subsequent mouse experiment. Mean percent survival in mouse and chicken mouse macrophage survival assays were also compared using Student’s *t* test. Mean inflammation score for each parameter as wells as mean combined inflammation score were compared for the entire experiment using two-way ANOVA with Tukey’s post hoc analysis to identify statistically significant differences (*P* < 0.05).

## Results and discussion

### SPI-13 contributes to colonization of the intestine and internal organs, and induction of acute cecal inflammation by *S.* Enteritidis in streptomycin pre-treated mice

NTS infection in the conventional laboratory mice typically manifests as a systemic disease resembling typhoid in humans (Reviewed in [18]). In contrast, pre-treatment with high dose of streptomycin (20 mg) 24 h prior to infection results in reduced diversity of the microflora in the gastrointestinal tract. This in turn, allows *Salmonella* to extensively colonize the intestinal tract resulting in gastro-intestinal disease that closely resembles human intestinal disease which is characterized by acute gut inflammation and diarrhea (Reviewed in [19]). Thus, streptomycin pre-treated mouse model has been extensively characterized and widely adapted as an improved model to assess the role of *Salmonella* genetic factors in causing enterocolitis [19].

**Fig. 2:**
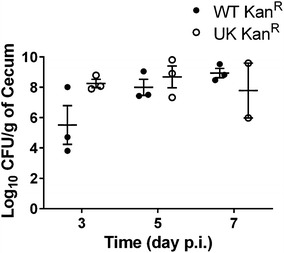
Pilot experiments determined that a dose of 7 log_10_ CFU resulted in optimal cecal colonization (CFU/g) of *S.* Enteritidis in a streptomycin pre-treated mouse model. C57BL/6 mice were infected with 7 log_10_ CFU of the WT Kan^R^ or UK Kan^R^ WT strains. *Bars* represent mean ± SEM of three mice for each time point except for day 7 p.i. where only two mice were sacrificed in the UK Kan^R^ infected group. Significant differences were determined with two-sample *t* test not assuming equal variances (*P < 0.05)

Most of the published studies have used aminoglycoside resistant *S.* Typhimurium as a model organism and have reported that a dose as low as ~2 log_10_ CFU is sufficient to efficiently colonize streptomycin pre-treated mice, however a dose of ~7 log_10_ CFU has been more commonly used [15, 19]. Limited number of studies have employed this model to study infection kinetics of aminoglycoside resistant *S.* Enteritidis also reported that this model can be used for this serotype as well [15, 20]. Because the WT strain (CDC_2010K_0968) used in the current study is not naturally resistant to aminoglycoside, we constructed an aminoglycoside resistant derivative of WT CDC_2010K_0968 strain (WT Kan^R^) by introducing kanamycin resistance cassette downstream of the tn7 attachment site (*att_tn7*, between *glmS* and *SEN3674*). This genomic site has been previously used for insertion of heterologous genes with no adverse effects on in vitro or in vivo phenotypes of WT *Salmonella* strains [21]. As expected, the WT Kan^R^ strain did not show any phenotypic differences when the in vitro growth kinetics were compared with the parental WT or with ΔSPI-13, suggesting that insertion of kanamycin cassette did not alter in vitro growth characteristics of these strains (data not shown). We also compared the kinetics of infection of WT Kan^R^ strain with that of *S.* Enteritidis P125109 strain (UK Kan^R^) which was originally isolated from a human food-borne outbreak in the United Kingdom [13] and has been used as a reference strain in published studies. As expected, both strains colonized cecum to similar levels in this model (Fig. 2), suggesting that the kinetics of infections of WT Kan^R^ strain are comparable to the widely used UK Kan^R^ as has been described previously [15, 20].

**Fig. 3:**
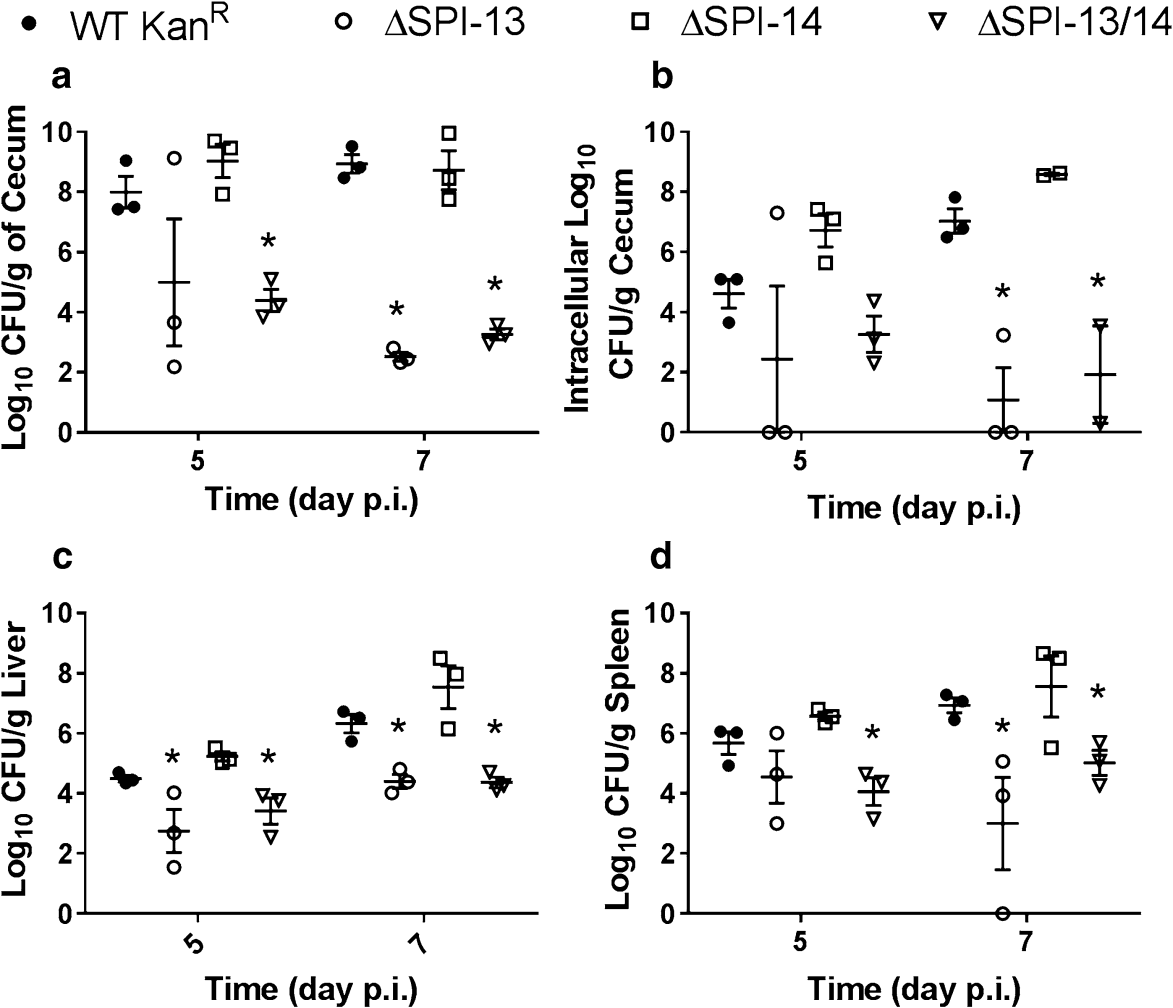
Deletion of SPI-13 but not SPI-14 results in impaired colonization of the cecum (**a**), reduced invasion of the intestinal epithelium (**b**), and impaired colonization of the liver (**c**) and spleen (**d**). *Bars* represent mean ± SEM of three mice for each time point except for day 7 p.i. where in the ΔSPI-13/14 infected group where mean intracellular cecal counts from two animals were used. Significant differences were determined using a one-way ANOVA with Tukey’s post hoc for multiple comparisons (*P < 0.05)

Subsequently, we compared the pathogenicity of the WT Kan^R^ and the ΔSPI-13 mutant. In this experiment, we also included ΔSPI-14 and ΔSPI-13/14 mutants that were constructed for an unrelated experiment, but served as additional controls to compare and confirm the role of SPI-13 in pathogenesis. Streptomycin pre-treated mice were challenged with ~7 log_10_ CFU of the WT Kan^R^, ΔSPI-13, ΔSPI-14, or ΔSPI-13/14 strain and the kinetics of infection were determined on days 5 and 7 p.i. On day 5 p.i., at least two out of three animals challenged with ΔSPI-13 showed lower bacterial counts in the cecum and other organs (Fig. 3). This trend was also observed in the animals challenged with ΔSPI-13/14 mutant, but not the ΔSPI-14 mutant. The effects of absence of SPI-13 became more evident on day 7 p.i. as the total as well as intracellular counts of the ΔSPI-13 as well as ΔSPI-13/14 mutants in the cecum were significantly (*P* < 0.05) lower than the WT Kan^R^. However the kinetics of infection with ΔSPI-14 remained similar to WT Kan^R^, suggesting that absence of SPI-13 significantly impaired the intestinal colonization and invasion of the intestinal epithelium (Fig. 3). Similarly, the counts of ΔSPI-13 and ΔSPI-13/14 were lower in liver and spleen when compared with the WT Kan-^R^ however only the differences in the spleen counts were statistically (*P* < 0.05) significant (Fig. 3), suggesting that ΔSPI-13 mutant was also impaired in its ability to colonize internal organs.

**Fig. 4:**
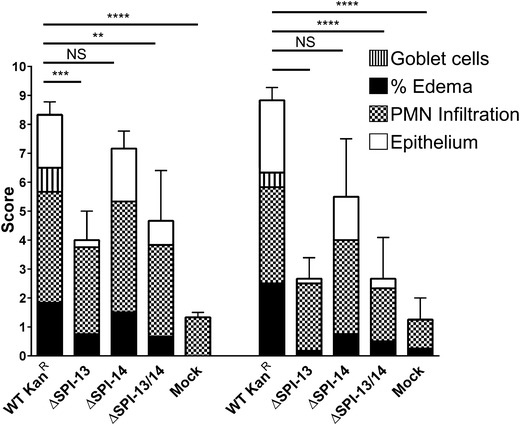
Deletion of SPI-13 but not SPI-14 results in reduced cecal inflammation in orally infected mice. *Bars* represent mean inflammation severity score ± SEM from three mice for each time point with the contribution from individual parameters indicated. Significant differences were determined using Tukey’s post hoc analysis of two-way ANOVA (*NS* not significant; **P < 0.01; ***P < 0.001; ****P < 0.0001)

**Fig. 5:**
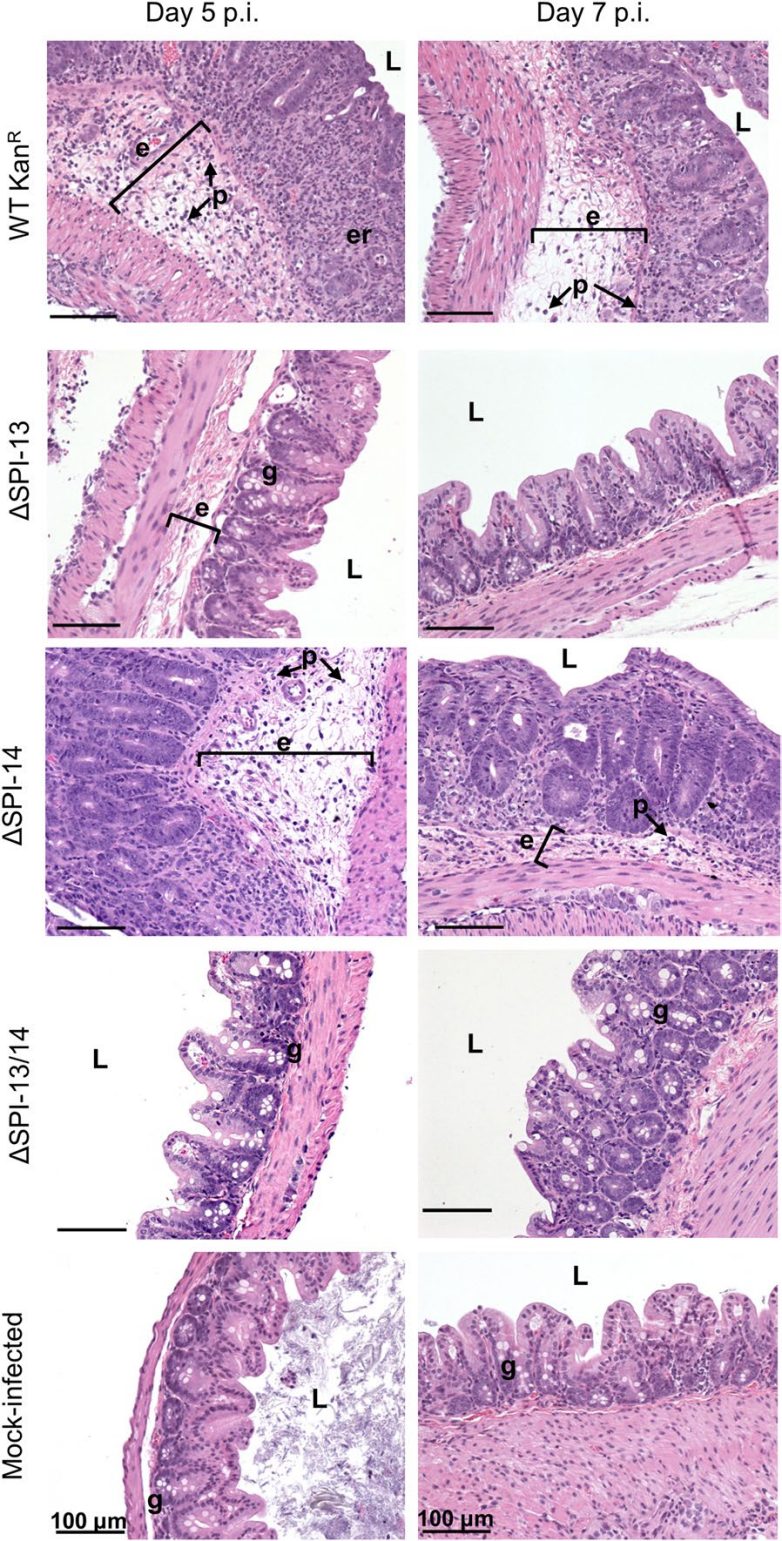
The WT Kan^R^ strain produces severe inflammation of the cecum characterized by epithelial erosion and necrosis (*er*), infiltration of PMNs (*p*), edema (*e*), and a reduction in the number of goblet cells (*g*) whereas the ΔSPI-13 mutant produces mild or more similar to the level of inflammation observed with mock-infected controls. Representative H&E stained cecal cross-sections are shown from each group on days 5 and 7 p.i. *Scale bar* indicates 100 μm. *L* lumen

In addition to the impaired intestinal and internal organ colonization, the combined cecal inflammation score, compromised of the sum of the indidivual scores for percent edema, infiltration of PMNs, loss of goblet cells, and epithelial integrity, was significantly (*P* < 0.05) lower on days 5 and 7 p.i. among animal groups infected with ΔSPI-13 and ΔSPI-13/14 mutant when compared with WT Kan-^R^, but the inflammation scores were not significantly different in animals challenged with ΔSPI-14 mutant (Figs. 4, 5). By day 7 p.i., the inflammation score for the WT Kan-^R^ and ΔSPI-14 challenged groups were significantly higher than mock infected control (Fig. 4). In contrast, the inflammation scores for the ΔSPI-13 and ΔSPI-13/14 challenged groups were not significantly different from the mock-infected controls (Fig. 4). Collectively, these data show that absence of SPI-13 results in the reduced intestinal and internal organ colonization and significantly reduced intestinal inflammation in streptomycin pre-treated mice. Additionally, infection kinetics of ΔSPI-13/14 were similar to ΔSPI-13 whereas infection kinetics of ΔSPI-14 were similar to the WT Kan-^R^ further confirming that SPI-13 indeed contributes to the pathogenesis of *S.* Enteritidis in streptomycin pre-treated mice.

### SPI-13 contributes to survival in mouse macrophages

**Fig. 6:**
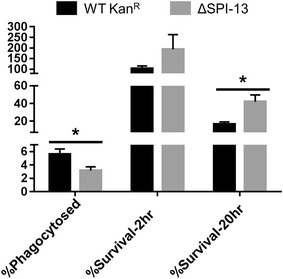
Deletion of SPI-13 results in increased uptake and reduced survival (2 and 20 h) in mouse RAW264.7 macrophages. *Bars* represent mean percent of each phenotype from three biological replicates ± SEM. Significant differences were determined using two-sample *t* test not assuming equal variances (*P < 0.05)

Uptake by and survival/replication within the host macrophages is a critical step for *Salmonella* to colonize the internal organs of hosts. *Salmonella* is specifically adapted to the intracellular niche and survives within macrophages by avoiding phago-lysosomal fusion through formation of the *Salmonella* containing vacuole (SCV). Infected macrophages then act as vehicle and transport *Salmonella* to the internal organs via the circulatory system. Therefore we tested the survival of the ΔSPI-13 mutant in mouse macrophages as this may relate to the reduced internal organ colonization shown in Fig. 3. Intra-macrophage survival assay showed that the ΔSPI-13 mutant (39.4 %) was more readily phagocytosed when compared with WT Kan^R^ (2.9 %). Moreover, the survival/replication rate of ΔSPI-13 (57.3 %) at 2 h p.i. was significantly (*P* < 0.05) lower than the WT Kan^R^ (609.6 %). Similarly, ΔSPI-13 (2.4 %) showed significantly (*P* < 0.05) lower survival at 20 h when compared with the WT Kan^R^ (24.3 %) (Fig. 6). These data corroborate with the previous report showing that deletion of a first gene of SPI-13 (*STM3117*) in *S.* Typhimurium resulted in impaired survival in murine macrophages [22]. Additionally, it has been previously reported that expression of several SPI-13 genes is upregulated in *S.* Typhimurium during intra-macrophage infection [23, 24]. Therefore, our data corroborates with the previous reports and shows that SPI-13 contributes to the intra-macrophage survival of *S.* Enteritidis. Thus, decreased colonization of internal organs by the ΔSPI-13 mutant could, in part, be attributed to its decreased systemic dissemination due to impaired survival in macrophages. Interestingly, deletion of *ripABC* operon, which is homologous to *SEN2961*–*SEN2963*, in *Y. pestis* is also associated with decreased survival in mouse macrophages [25], further suggesting that SPI-13 is important for efficient replication in intracellular environment of phagocytic cells. However, the reports of potential mechanisms underlying for intra-macrophage survival are contradictory. Torres and others [26] postulated that *rip* operon of *Y. pestis* is likely responsible for production of butyrate, a molecule that has anti-inflammatory properties. These authors suggested that butyrate reduces the production of nitric oxide by macrophages and thereby enhances intra-macrophage survival of *Y. pestis*. In contrast, a more recent biochemical characterization of Rip proteins of *Y. pestis* showed that these proteins are more likely involved in degradation of itaconate [27]. Itaconate is is produced by macrophages in response to immune responsive gene 1 (*IRG1*) expression and recognized as a potent antimicrobial which inhibits the glyoxylate cycle of bacteria [28]. We performed bioinformatics analysis of SPI-13 genes and found that the first ten genes in SPI-13 (*SEN2960*–*SEN2969*) are predicted to encode putative proteins that are likely involved in the metabolism of aromatic monoamines or related molecules such as itaconate and the last gene (*SEN2977*) is a putative hexuronate transporter which is likely involved in uptake or hexuronate, suggesting that SPI-13 is likely involved in metabolism of multiple metabolic substrates. Although intriguing, it is currently unknown which of these metabolic mechanisms play an active role during *S.* Enteritidis infection in intestinal and intracellular environment in mice. More research is needed to improve our mechanistic understanding of role of SPI-13 in *Salmonella* metabolism and in particular its link to intestinal infection and intra-macrophage survival of *S.* Enteritidis.

### *Salmonella* Enteritidis SPI-13 does not significantly impair the intestinal colonization and organ colonization in chickens

**Fig. 7:**
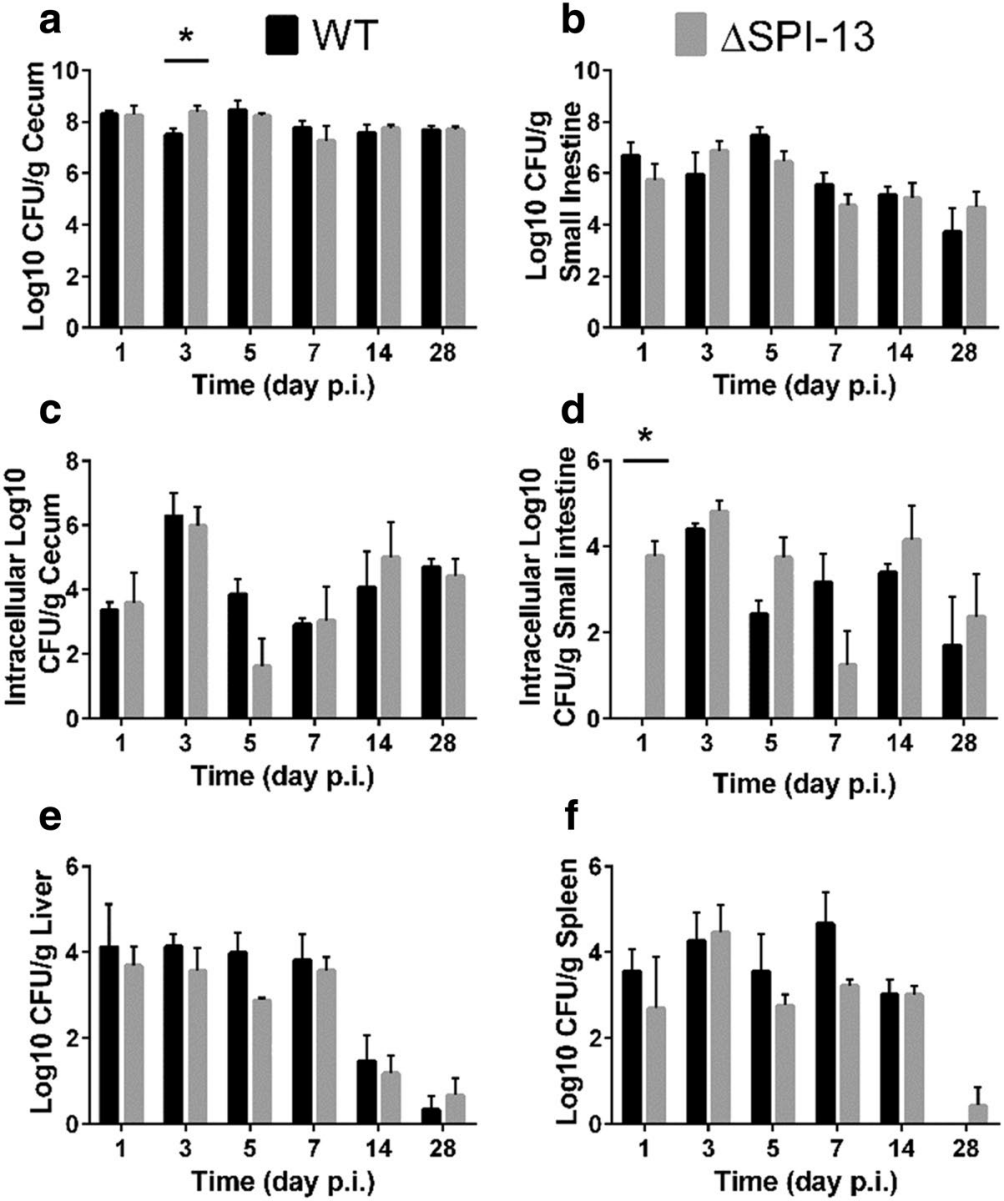
Deletion of SPI-13 does not significantly impact pathogenesis of *S.* Enteritidis in chickens. One-day-old chickens were orally infected with 8.5 log_10_ CFU of WT (*black bars*) or ΔSPI-13 mutant (*gray bars*). Mean total log_10_ CFU/g of tissue in the cecum (**a**), small intestine (**b**), liver (**c**), spleen (**d**), and mean intracellular log_10_ CFU/g of both strains in the cecum (**e**) and small intestine (**f**). Each time point represents the mean + SEM of four chickens. Significant differences (*P* < 0.05) were identified using two-sample *t* test not assuming equal variance for each time point

We were particularly interested in the contribution of SPI-13 to the pathogenesis in chickens because poultry and poultry products have been implicated as the primary vehicles for the transmission of *S.* Enteritidis to people [29, 30]. Therefore, we infected two groups of 24, 1-day-old chickens with ~8.5 log_10_ CFU of the WT parent or the ΔSPI-13 mutant strain and determined the kinetics of infection in the liver, spleen, cecum, and small intestine up to 28 days p.i. The results revealed that the total and intracellular counts in both cecum and small intestine of chickens infected with ΔSPI-13 were not significantly different from the WT infected chickens (Fig. 7). Note that on day 1 p.i., sample-processing error resulted in residual gentamicin in the small intestinal samples from chickens challenged with WT resulting in the killing of the bacteria released after cell lysis and subsequent negative isolation results. Similarly, we found no significant differences in the colonization of spleen and liver between ΔSPI-13 mutant and WT parent strain (Fig. 7). Interestingly, two chickens challenged with WT strain died during the course of experiment (1 on day-1 p.i. and 1 on day-4 p.i.); however no mortality was observed in chickens inoculated with the ΔSPI-13 mutant strain. Collectively, the results show that absence of SPI-13 did not significantly impair the ability of *S.* Enteritidis to colonize the intestine and internal organs of chickens, however the difference in mortality raises a possibility that absence of SPI-13 may have resulted in subtle reduction of virulence of *S.* Enteritidis in day-old chickens. Determination of the LD50 of the ΔSPI-13 mutant will be needed to confirm and conclusively demonstrate these differences in virulence in chicken. Noteworthy is that our results contradict the previous report in which *S.* Typhimurium mutants with insertion mutations within several SPI-13 genes were negatively selected in orally inoculated chickens [8]. There are multiple plausible explanations for this discrepancy. First, we employed individual strain infections whereas Chaudhuri et al. [8] employed mixed infection approach in which reduced competitive fitness of the mutants could result in their negative selection. It is also possible that SPI-13 mutants reported by Chaudhuri et al. [8] could represent false positives because a high false discovery rate of negatively selected mutants was reported by these authors. Serotype differences could also contribute to discrepancies between our results and previous studies because *S.* Typhimurium and *S.* Enteritidis differ in their repertoire of pathogenicity factors [11, 31]. The apparent role of SPI-13 in metabolism could also explain the difference in phenotypes observed in SPI-13 mutants of *S.* Enteritidis in this study and *S.* Gallinarum in our previous study [6]. *Salmonella* Gallinarum is known to have more pseudogenes than *S.* Enteritidis and several of the genes encoding metabolic functions are degraded in this host-adapted serotype making this serotype metabolically auxotrophic [13]. Consequently, it is possible that SPI-13 genes partly fulfil the metabolic requirements of *S.* Gallinarum in chicken host and therefore its absence may adversely affect infectivity of *S.* Gallinarum in chickens, but does not affect infectivity of metabolically more efficient *S.* Enteritidis in the same host. Further biochemical characterization of ΔSPI-13 will be required to identify exact target metabolic substrates for genes encoded on this island. Research is also needed to determine the potential role of such metabolic substrates in vivo during *Salmonella* infection streptomycin pre-treated mouse.

### SPI-13 does not contribute to the survival of *S*. Enteritidis in chicken macrophages

**Fig. 8:**
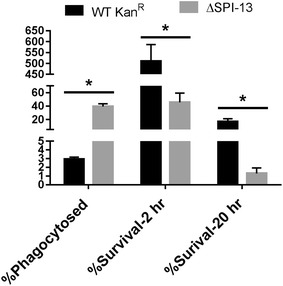
Deletion of SPI-13 does not affect survival in HD11 chicken macrophages. Macrophages were infected at an MOI of ~20 and the mean percent uptake and percent survival at 2 and 20 h post infection was determined from three biological replicates. Significant differences were determined using two-sample *t* test not assuming equal variances (*P < 0.05)

Previously, transposon insertion mutation in *SEN2961* was reported to result in impaired intra-cellular survival of *S.* Enteritidis in avian macrophages [32]. Consequently, we tested the intra-macrophage survival of the ΔSPI-13 mutant strain and compared with the WT parent strain using chicken macrophage-like cells (HD11). Interestingly, the uptake (30 min) of ΔSPI-13 in HD-11 cells was higher than its WT counterpart (Fig. 8). However, in contrast to the previous report [32], the intracellular survival of ΔSPI-13 mutant was similar to WT at early (2 h) and slightly higher than WT at late (20 h) intracellular phase (Fig. 8). These data suggest that the absence of SPI-13 does not result in impaired intra-macrophage survival of *S.* Enteritidis.

## Conclusions

In summary, our results show that SPI-13 contributes to the pathogenesis of *S.* Enteritidis which is characterized by decreased colonization of the cecum, reduced inflammation and reduced colonization of the spleen and liver in streptomycin pre-treated mice. The decreased colonization may be at least in part due to the decreased survival in murine macrophages that could limit dissemination to the internal organs. In contrast, we did not observe significant difference in the pathogenesis of ΔSPI-13 mutant in chicken host and there was no defect in the growth and/or survival of the mutant in chicken macrophages. These data raise several interesting questions why SPI-13 is required for full virulence of *S.* Enteritidis in murine host, but does not appear to be imporant in its reservoir host, chickens? Is it is likely that SPI-13 is a host-specific pathogenicity island? and if true, is this host-specificity is linked to the potential role of SPI-13 in *S.* Enteritidis metabolism which may impact metabolic fitness of *S.* Enteritidis in specific hosts? It is intriguing because broad-host range *Salmonella* serovars are known to have diverse metabolism to support optimal growth in potentially nutrient-limited conditions encountered in different hosts or in the external environment [33]. One plausible hypothesis is that SPI-13 may be important for *S.* Enteritidis’s ability to utilize monoamines and/or hexuronates in hosts such as streptomycin pre-treated mouse where these nutrients may serve as primary source of energy in vivo. In contrast, this nutrient demand of *S.* Enteritidis in chicken host is compensated with other sources making SPI-13 dispensable in this reservoir host.

Finally, several *Salmonella* serovars such as Typhi, Paratyphi A and Sendai and at least few NTS serotypes, have a different SPI-13 gene composition compared to the majority of the other NTS serovars with broad host range [6, 7]. More specifically, the first six genes within SPI-13 are completely absent in *S.* Typhi and *S.* Paratyphi whereas *S.* Seftenberg and *S.* Infantis have large insertions at the upstream end of SPI-13. This suggest that SPI-13 has a modular architechture [6, 7]. However it is currently unknown if this genetic diversity within SPI-13 also contributes to differential virulence of *Salmonella* in different hosts and if it is associated with differential of metabolic dependence of *Salmonella* in different hosts. The modular architechture of SPI-13 is intriguing. We chose to use the most inclusive description of SPI-13 for defining SPI-13 in the current study. However, work is in progress to more rigorously define the island and determine which genes within this island specifically contribute to the pathogenesis and metabolism of *S.* Enteritidis.
