# Erratum to: The Salmonella pathogenicity island 13 contributes to pathogenesis in streptomycin pre-treated mice but not in day-old chickens

**DOI:** 10.1186/s13099-016-0114-4

**Published:** 2016-07-12

**Authors:** Jacob R. Elder, Kim Lam Chiok, Narayan C. Paul, Gary Haldorson, Jean Guard, Devendra H. Shah

**Affiliations:** Department of Veterinary Microbiology and Pathology, Washington State University, Pullman, WA 99164-7040 USA; Paul Allen School for Global Animal Health, College of Veterinary Medicine, Washington State University, Pullman, WA 99164-7040 USA; Egg Quality and Safety Research Unit, Agriculture Research Service, United States Department of Agriculture, Athens, GA 30605 USA

## Erratum to: Gut Pathog (2016) 8:16 DOI 10.1186/s13099-016-0098-0

Following publication of the original article in *Gut Pathogens* [1], it was brought to our attention that graphs for Figures 6 and 8 were interchanged and are therefore incorrect.

Please find the correct Figs. 6 and 8 below. Also in the Results and Discussion section titled “SPI-13 does not contribute to the survival of S. Enteritidis in chicken macrophages,” the uptake of the ΔSPI-13 mutant is incorrectly described as having a higher uptake in HD11 macrophages compared to the WT strain. We observed higher uptake of the WT strain in HD-11 cells.

**Fig. 6:**
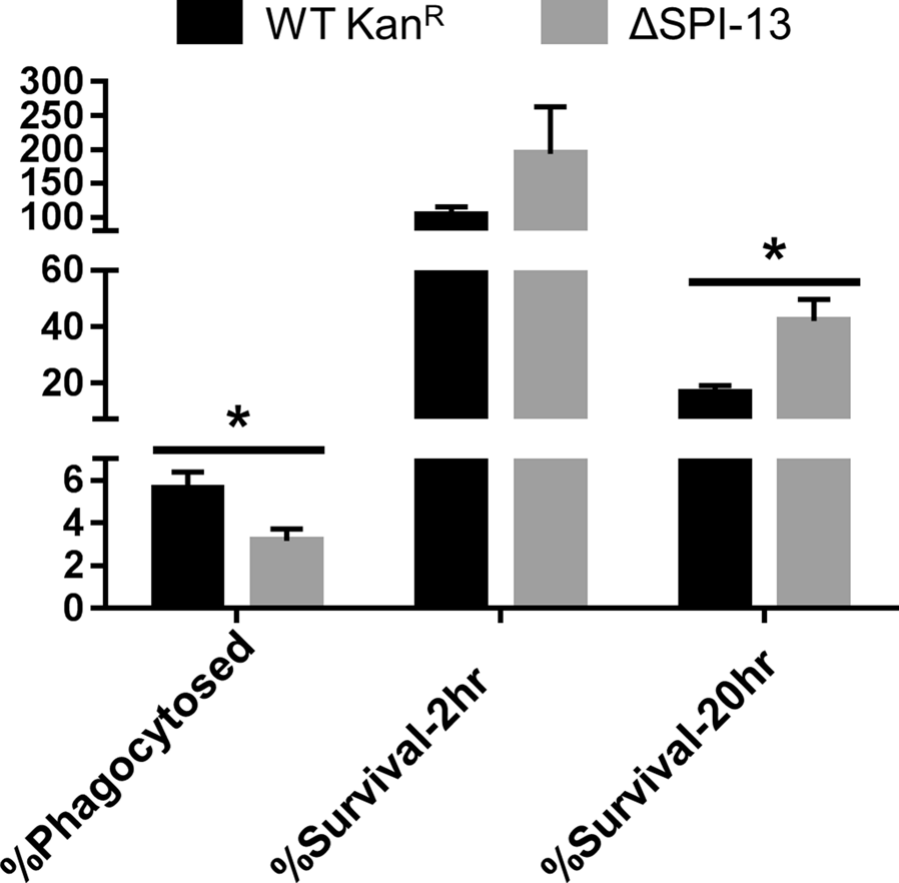
Deletion of SPI-13 results in increased uptake and reduced survival (2 and 20 h) in mouse RAW264.7 macrophages. *Bars* represent mean percent of each phenotype from three biological replicates ± SEM. Significant differences were determined using two sample* t* test not assuming equal variances (*P < 0.05)

**Fig. 8:**
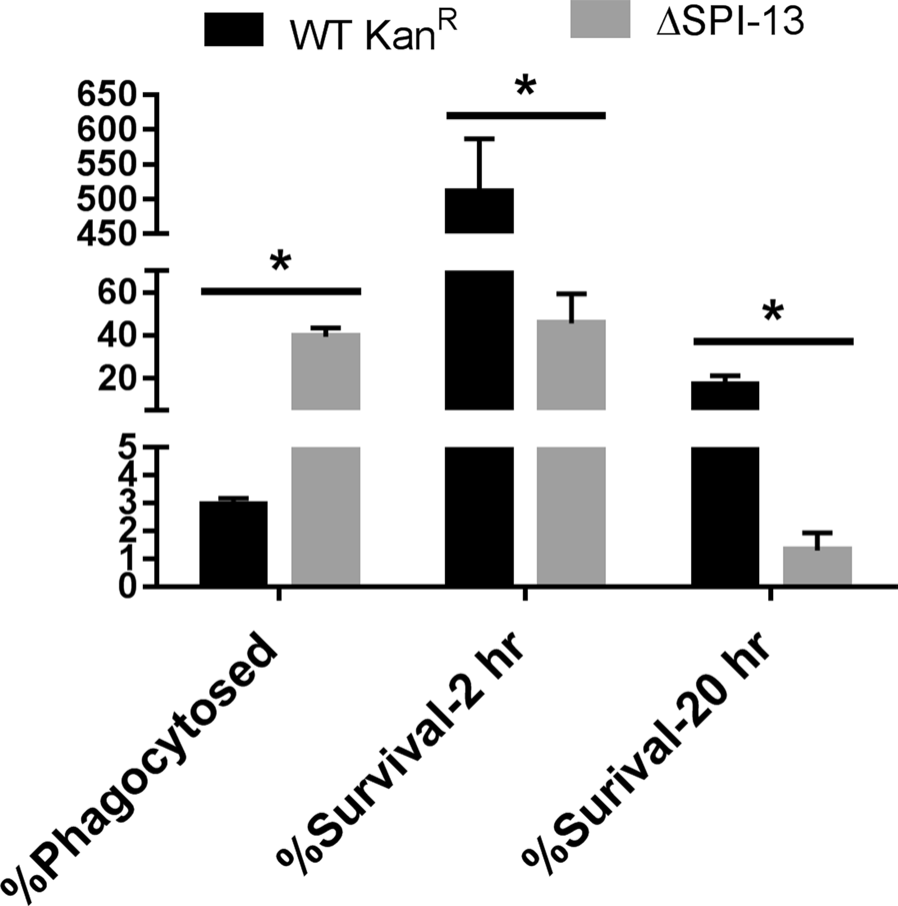
Deletion of SPI-13 does not affect survival in HD11 chicken macrophages. Macrophages were infected at an MOI of ~20 and the mean percent uptake and percent survival at 2 and 20 h post infection was determined from three biological replicates. Significant differences were determined using two-sample* t* test not assuming equal variances (*P < 0.05)

We apologize for the inconvenience this may have caused.
